# Automated Incubation and Digital Image Analysis of Chromogenic Media Using Copan WASPLab Enables Rapid Detection of Vancomycin-Resistant Enterococcus

**DOI:** 10.3389/fcimb.2019.00379

**Published:** 2019-11-06

**Authors:** Abdessalam Cherkaoui, Gesuele Renzi, Yannick Charretier, Dominique S. Blanc, Nicolas Vuilleumier, Jacques Schrenzel

**Affiliations:** ^1^Bacteriology Laboratory, Division of Laboratory Medicine, Department of Diagnostics, Geneva University Hospitals, Geneva, Switzerland; ^2^Genomic Research Laboratory, Division of Infectious Diseases, Department of Medical Specialities, Faculty of Medicine, Geneva, Switzerland; ^3^Service of Hospital Preventive Medicine, Lausanne University Hospital, Lausanne, Switzerland; ^4^Swiss National Reference Center for Emerging Antibiotic Resistance (NARA), Fribourg, Switzerland; ^5^Division of Laboratory Medicine, Department of Diagnostics, Geneva University Hospitals, Geneva, Switzerland; ^6^Division of Laboratory Medicine, Department of Medical Specialities, Faculty of Medicine, Geneva, Switzerland

**Keywords:** Copan WASPLab, time to results, vancomycin-resistant *Enterococcus*, VRE, culture, screening, chromogenic media

## Abstract

**Objective:** The aim of the present study was to assess whether the WASPLab automation enables faster detection of vancomycin-resistant *Enterococcus* (VRE) on chromogenic VRE-specific plates by shortening the incubation time.

**Methods:** Ninety different VRE culture negative rectal ESwab specimens were spiked with various concentrations (ranging from 3 × 10^2^ to 3 × 10^7^ CFU/ml) of 10 *Enterococcus faecium* strains (vancomycin MICs ranging from 32 to >256 mg/l), 3 *E. faecium* VanB strains (vancomycin MICs: 4, 8, and 16 mg/l), and 2 *E. faecium* VanB strains displaying vancomycin heteroresistance (vancomycin MICs: 64 and 96 mg/l).

**Results:** Besides the two strains exhibiting vancomycin heteroresistance, all the other 13 VRE strains included in this study were detected as early as 24 h on the WASPLab even if the inoculum was low (3 × 10^3^ CFU/ml). When the vancomycin MICs were high, all strains were detected as early as at 18 h. However, 30 h was a conservative time point for finalizing the analysis of chromogenic cultures.

**Conclusion:** These results suggested that the WASPLab automated incubation could allow decreasing the initial incubation time to 18 h, followed by an intermediate time at 24 h and a final incubation period of 30 h for VRE culture screening, to deliver rapid results without affecting the analytical sensitivity.

## Introduction

Since the 1970's, enterococci are considered as one of the common causes of hospital-acquired infections. Several reasons have been advanced to explain why these pathogens are frequently isolated in the hospital environment, including their capacity to acquire resistance to almost all available antibiotics and their ability to survive for long periods on surfaces (Kramer et al., 2006; Garcia-Solache and Rice, 2019). Enterococci are commensal colonizers of the gastrointestinal tract but they are involved in a variety of serious nosocomial infections, such as urinary tract infections, intraabdominal infections, bacteremia and endocarditis (O'Driscoll and Crank, 2015; Shah et al., 2018). Until the late eighties, vancomycin was considered as the only effective antibiotic for the treatment of infections associated with multidrug-resistant enterococci. This situation was challenged by the report of the first case of a vancomycin resistant *Enterococcus* (VRE) infection in 1988 (Uttley et al., 1988). Vancomycin resistance is caused by the presence of the van operon, carried on either a plasmid or the chromosome, and appears to be inducible (Ahmed and Baptiste, 2018). Nowadays, VRE are widespread and their prevalence can reach up to 35% of all enterococci isolated, contingent upon the geographical location (Werner et al., 2008; O'Driscoll and Crank, 2015). Transmission from surface to person or from person-to-person is a severe threat since the infections caused by VRE have been linked to an increased mortality rate (Diazgranados et al., 2005). The early identification of colonized patients by active screening of high-risk patients remains the effective way to prevent transmission, by enabling timely implementation of adapted infection control measures and surveillance, especially for vancomycin-resistant *vanA/vanB* types *Enterococcus faecium* and *Enterococcus faecalis* (Vehreschild et al., 2018). Nowadays, the majority of clinical microbiology laboratories use chromogenic media to screen for the presence of VRE in fecal or rectal swab specimens. However, plating and interpreting bacterial growth on chromogenic VRE plates can be time-consuming.

The main objective of this study was to assess whether the use of the WASPLab automation allows shorter incubation times of specific chromogenic plates and permits earlier identification of colonized patients, as well as earlier confirmation of the absence of carriage, both helping to prevent transmission and to reduce unnecessary cohorting.

## Materials and Methods

### Bacterial Strains

The 15 vancomycin*-*resistant *E. faecium* strains examined in this study were taken from a collection of non-consecutive clinical isolates identified at Geneva university hospitals (HUG) and by the Swiss National Reference Centre for Emerging Antibiotic Resistance (NARA). Table 1 depicts the characteristics of these clinical strains. Vancomycin MICs were determined by the gradient strip method (Etest, bioMérieux Marcy l'Etoile, France) according to the manufacturer's instructions. Importantly, the gradient strip method (Etest) or MIC Test Strip (Liofilchem, Italy) underestimates the vancomycin MIC for *E. faecalis* and *E. faecium* strains carrying the *vanA/vanB* genes but exhibiting low-level vancomycin resistance (Eucast, 2018). The identification of the strains was systematically confirmed by matrix-assisted desorption ionization time-of-flight mass spectrometry (MALDI-TOF MS; compass, Bruker Daltonics, Bremen, Germany) according to the manufacturer's instructions.

**Table 1:** Characteristics of the *Enterococcus faecium* strains used in this study.

**Strain ID, year of isolation, patient gender, patient age**	**VAN MIC by E-test**	**VAN MIC by broth micrcodilution**	**VAN (Disk diffusion 5 μg)**	**Growth on BHIA + 6 mg/l VAN**	**Teicoplanin MIC by E-test**	**Daptomycin MIC by E-test**	**Linezolid MIC by E-test**
*E. faecium* VanB (NARA-89)	6 (R)	4 (S)	R^*^	+	0.38 (S)	1 (S)	0.75 (S)
*E. faecium* VanB (NARA-388)	6 (R)	16 (R)	R^*^	+	1.5 (S)	2 (S)	0.75 (S)
*E. faecium* VanB (NARA-490)	32 (R)^**^	64 (R)	R^*^	+	1 (S)	1.5 (S)	1 (S)
*E. faecium* VanB (NARA-491)	96 (R)^**^	>64 (R)	R^*^	+	1 (S)	2 (S)	1 (S)
*E. faecium* VanB (NARA-492)	4 (S)	8 (R)	R^*^	+	0.75 (S)	3 (S)	0.75 (S)
*E. faecium* (2018, Female, 85 y) HUG	256 (R)	>64 (R)	R	+	48 (R)	3 (S)	1 (S)
*E. faecium* (2018, Female, 68 y) HUG	256 (R)	>64 (R)	R	+	1.5 (S)	3 (S)	1 (S)
*E. faecium* (2018, Female, <1 y) HUG	256 (R)	>64 (R)	R	+	12 (R)	2 (S)	0.75 (S)
*E. faecium* (2018, Female, <1 y) HUG	>256 (R)	>64 (R)	R	+	24 (R)	3 (S)	1 (S)
*E. faecium* (2018, Male, 30 y) HUG	32 (R)	64 (R)	R	+	1 (S)	2 (S)	1 (S)
*E. faecium* (2018, Male, <1 y) HUG	256 (R)	>64 (R)	R	+	16 (R)	3 (S)	1 (S)
*E. faecium* (2018, Male, 58 y) HUG	>256 (R)	>64 (R)	R	+	96 (R)	4 (S)	0.5 (S)
*E. faecium* (2018, Male, 84 y) HUG	128 (R)	>64 (R)	R	+	1 (S)	2 (S)	0.75 (S)
*E. faecium* (2018, Male, 90 y) HUG	48 (R)	>64 (R)	R	+	0.75 (S)	2 (S)	0.75 (S)
*E. faecium* (2018, Female, 7 y) HUG	>256 (R)	>64 (R)	R	+	64 (R)	8 (R)	0.75 (S)

*VAN, Vancomycin; MIC, Minimum Inhibitory Concentration*.

* *Fuzzy zone edges*,

** *Presence of a resistant heteropopulation*.

*R, resistant; S, susceptible*.

*Daptomycin MICs were interpreted according to CLSI breakpoints*.

### Preparation of the Spiked Samples

As the VRE prevalence in our institution is very low (about 1% of all isolated *Enterococcus)*, the specimens of this study were constituted of 90 different VRE negative rectal ESwab (Copan, Brescia, Italy) specimens spiked with various concentrations (ranging from 3 × 10^2^ to 3 × 10^7^ CFU/ml) of the 15 VRE strains included in this study. The final density was systematically controlled by viable cell counting. We checked also if the CFUs we retrieved on selective media corresponded to the actual CFUs spiked.

The rectal ESwabs used in this study were referred to the bacteriology laboratory at HUG, and validated as VRE negative by culture. The chromogenic agar used in this study was the chromID^®^ VRE (bioMérieux Marcy l'Etoile, France) that contains 8 mg/l of vancomycin. Chromogenic VRE plates were inoculated with 30 μl of negative rectal ESwabs specimens spiked with defined VRE concentrations ranging from 3 × 10^2^ to 3 × 10^7^ CFU/ml for each of the 15 VRE strains analyzed in this study.

### Sample Processing

In the absence of any enrichment, we determined the shortest incubation times for (i) detecting, and (ii) ruling out the presence of VRE, respectively, by performing time-series image acquisition on the WASPLab (Copan WASP srl, Brescia Italy) at sequential time points (18, 24, 30, 36, 40, and 48 h). For each evaluated incubation time point, several high-resolution digital images were acquired under different light and exposure conditions according to manufacturer's instructions. Growth of VRE was defined by the presence of the specific VRE color on chromID^®^ VRE, and was systematically quantified on the WASPLab. Finally, we assessed the results obtained by WASPLab automation against those obtained by WASP-based inoculation followed by conventional incubation and manual diagnostic which constitutes the routine method used in our laboratory.

### Population Analysis Profiling

Population analysis profiling (PAP) was performed using a modified spotting method (Pfeltz et al., 2001). Briefly, a suspension of each strain was adjusted to 2.0 McFarland and serially 10-fold diluted to 10^−7^. All suspensions were spotted using 10 μl droplets on Mueller Hinton agar and on plates containing serial dilutions of vancomycin at concentrations from 64 to 0.25 mg/l. Two replicates were performed. Colonies were counted after 24 h of incubation at 37°C. A strain was identified as heteroresistant when the antibiotic exhibiting the highest inhibitory effect was >8-fold higher than the highest non-inhibitory concentration (El-Halfawy and Valvano, 2015).

## Results

As shown in Table 2, all the 60 negative rectal ESwabs spiked with increasing concentrations of the 10 HUG VRE strains (ranging from 3 × 10^2^ to 3 × 10^7^ CFU/ml) were already detected positive at 18 h when the chromogenic media was incubated on the WASPLab. No difference was observed between 18, 24, 30, 36, 40, and 48 h for both, the identification and the quantification of VRE, despite expected differences in the intensity of the specific color and the size of the colonies. All these 10 VRE strains were detected at 24 h by using WASP-based automated inoculation coupled to conventional incubation and the manual diagnostic method (Table 2).

**Table 2:** Results of the detection of the 15 vancomycin-resistant *Enterococcus faecium* strains on chromID^®^ VRE at different incubation time points on the WASPLab compared to WASP-based automated inoculation coupled to conventional incubation and manual diagnostic.

**Strain ID (vancomycin MIC)**	**Negative rectal ESwabs spiked by (CFU/ml)**	**Approx. cells inoculated on chromID^^®^^VRE (CFU)**	**WASP coupled to conventional incubation and manual diagnostic**		**WASPLab**					
			**Incubation time points**		**Incubation time points**					
			**24 h**	**48 h**	**18 h**	**24 h**	**30 h**	**36 h**	**40 h**	**48 h**
NARA-89 (4 mg/l)	3.00E + 07	1.00E + 06	+	+	+	+	+	+	+	+
	3.00E + 06	1.00E + 05	+	+	+	+	+	+	+	+
	3.00E + 05	1.00E + 04	+	+	+	+	+	+	+	+
	3.00E + 04	1.00E + 03	**–**	+	**–**	+	+	+	+	+
	3.00E + 03	1.00E + 02	**–**	+	**–**	+	+	+	+	+
	3.00E + 02	1.00E + 01	**–**	+	**–**	**–**	+	+	+	+
NARA-388 (16 mg/l)	3.00E + 07	1.00E + 06	+	+	+	+	+	+	+	+
	3.00E + 06	1.00E + 05	+	+	+	+	+	+	+	+
	3.00E + 05	1.00E + 04	+	+	+	+	+	+	+	+
	3.00E + 04	1.00E + 03	**–**	+	**–**	+	+	+	+	+
	3.00E + 03	1.00E + 02	**–**	+	**–**	+	+	+	+	+
	3.00E + 02	1.00E + 01	**–**	+	**–**	**–**	+	+	+	+
NARA-490 (64 mg/l)	3.00E + 07	1.00E + 06	+	+	+	+	+	+	+	+
	3.00E + 06	1.00E + 05	+	+	+	+	+	+	+	+
	3.00E + 05	1.00E + 04	**–**	+	+	+	+	+	+	+
	3.00E + 04	1.00E + 03	**–**	+	**–**	**–**	+	+	+	+
	3.00E + 03	1.00E + 02	**–**	+	**–**	**–**	+	+	+	+
	3.00E + 02	1.00E + 01	**–**	**–**	**–**	**–**	**–**	**–**	**–**	**–**
NARA-491 (96 mg/l)	3.00E + 07	1.00E + 06	+	+	+	+	+	+	+	+
	3.00E + 06	1.00E + 05	+	+	+	+	+	+	+	+
	3.00E + 05	1.00E + 04	+	+	+	+	+	+	+	+
	3.00E + 04	1.00E + 03	+	+	+	+	+	+	+	+
	3.00E + 03	1.00E + 02	**–**	+	**–**	**–**	+	+	+	+
	3.00E + 02	1.00E + 01	**–**	+	**–**	**–**	+	+	+	+
NARA-492 (8 mg/l)	3.00E + 07	1.00E + 06	+	+	+	+	+	+	+	+
	3.00E + 06	1.00E + 05	**–**	+	**–**	+	+	+	+	+
	3.00E + 05	1.00E + 04	**–**	+	**–**	+	+	+	+	+
	3.00E + 04	1.00E + 03	**–**	+	**–**	+	+	+	+	+
	3.00E + 03	1.00E + 02	**–**	+	**–**	+	+	+	+	+
	3.00E + 02	1.00E + 01	**–**	**–**	**–**	**–**	**–**	**–**	**–**	**–**
The 10 HUG VRE strains (MICs range 32 – >256 mg/l)	3.00E + 07	1.00E + 06	+	+	+	+	+	+	+	+
	3.00E + 06	1.00E + 05	+	+	+	+	+	+	+	+
	3.00E + 05	1.00E + 04	+	+	+	+	+	+	+	+
	3.00E + 04	1.00E + 03	+	+	+	+	+	+	+	+
	3.00E + 03	1.00E + 02	+	+	+	+	+	+	+	+
	3.00E + 02	1.00E + 01	+	+	+	+	+	+	+	+

**Figure 1:**
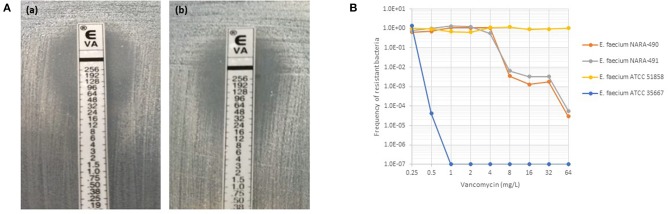
**(A)** Etest results for NARA-490 (a) and NARA-491 (b) strains with satellite colonies growing in the inhibition zone of vancomycin. **(B)** Vancomycin population analysis profiles for a fully susceptible (ATCC 35667), fully resistant (ATCC 51858), and heteroresistant strains (NARA-490 and NARA-491) to vancomycin.

As depicted in Figure 1A, NARA-490 and NARA-491 showed the presence of distinct colonies growing within the inhibition zone of the vancomycin indicating the presence of hetero-resistant subpopulations. Hetero-resistance to vancomycin was confirmed by population analysis profiles (Figure 1B). The NARA-490 strain was detected positive at 18 h on the WASPLab when the inoculum was equal or above 3 × 10^5^ CFU/ml. In contrast, when the inoculum was <3 × 10^5^ CFU/ml, NARA-490 was detected positive at 30 h on the WASPLab but only at 48 h when incubating the chromID^®^ VRE plates using a conventional incubator. Importantly, no growth was observed when the inoculum was equal to or <3 × 10^2^ CFU/ml, even after 48 h of incubation on the WASPLab or using the conventional incubator (Table 2). NARA-491 was detected positive at 18 h on the WASPLab when the inoculum was equal or above 3 × 10^4^ CFU/ml and only at 30 h when the inoculum was lower than 3 × 10^4^ CFU/ml (Table 2). NARA-492 (vancomycin MIC = 8 mg/l) was detected positive at 24 h on WASPLab but not at 18 h when spiked at concentrations equal to or <3 × 10^6^ CFU/ml. NARA-89 and NARA-388 were detected at 24 h of incubation on the WASPLab as compared to 48 h by using WASP coupled to conventional incubation and manual diagnostic. However, when the inoculum was equal to or higher than 3 × 10^5^ CFU/ml, the two strains were already detected at 18 h.

## Discussion

The most prevalent glycopeptide resistance genotypes in enterococci are *vanA* and *vanB* types. The increasing prevalence of *E. faecium* strains resistant to vancomycin is linked to the increasing rates of VRE in many European countries, with rates ranging between <1% in the Nordic European countries to more than 30% in Greece and Ireland (Werner et al., 2008). In Switzerland, several hospitals have reported nosocomial VRE outbreaks in the last few years (Thierfelder et al., 2012; Moulin et al., 2018; Abdelbary et al., 2019; Niccolò et al., 2019). In the United States, 35.5% of all *Enterococcus* isolated in health care-acquired infections are resistant to vancomycin (Sievert et al., 2013).

To prevent VRE outbreaks, screening of patients at risk and implementation of isolation precaution is recommended by different guidelines (Siegel et al., 2007; Frakking et al., 2018). Screening relies on culture using chromogenic VRE plates coupled (or not) with the detection of *vanA* or *vanB* genes by PCR. According to different published PCR-based screening for VRE assays, the sensitivity for the detection of *vanA* gene ranged from 43.5 to 100% and the specificity ranged from 79.2 to 99.6%. However, the detection of the *vanB* gene has very low specificity, putatively due to the presence of the *vanB* gene in anaerobic bacteria (Mak et al., 2009; Werner et al., 2011; Holzknecht et al., 2017). Many routine clinical laboratories use different systems for PCR-based screening for VRE (e.g., GeneXpert, Amplex…). Nowadays these systems are still marginally more expensive than cultures.

Besides the two strains exhibiting vancomycin heteroresistance, all the other 13 VRE strains included in this study were detected as early as 24 h on the WASPLab even if the inoculum was low (3 × 10^3^ CFU/ml), confirming that 30 h was a conservative time point for finalizing the analysis of chromogenic cultures. However, when the vancomycin MICs were high, all strains were detected as early as at 18 h, although the bacterial growth was lower when compared to 24 h of incubation.

The detection of VRE strains exhibiting low vancomycin MICs is more challenging. However, our results highlight that the WASPLab coupled to chromogenic media enables rapid diagnosis of VRE, by detecting the presence or conversely ruling out the carriage at 24 and 30 h, respectively. Overall, for all the 15 different VRE strains included in this study, automation permitted substantial reduction of the incubation time without affecting the analytical sensitivity as compared to WASP-based automated inoculation coupled to conventional incubation and manual diagnostic.

The potential limitation of this study relates to the fact that we analyzed spiked negative rectal ESwabs, but we managed to include a large range of vancomycin MICs (from 4 up to >256 mg/l), including locally prevalent strains and heteroresistant strains, to assess the performances using serial dilutions. Further studies using large series of clinical samples are still required.

## Conclusion

Overall, this study shows that the use of the WASPLab coupled to a VRE-chromogenic medium reliably decreases the time before the availability of VRE culture results. Our results suggest to define an early incubation time at 18 h, an intermediate time at 24 h and a final incubation period of 30 h for VRE culture screening, to prevent transmission and to reduce unnecessary cohorting.

## Data Availability Statement

The raw data supporting the conclusions of this manuscript will be made available by the authors, without undue reservation, to any qualified researcher.

## Ethics Statement

In accordance with local ethical committee, routine clinical laboratories of our institution may use biological sample leftovers for method development after irreversible anonymization of data. The official name of the ethics committee is Commission cantonale d'éthique de la recherche (CCER). https://www.hug-ge.ch/ethique.

## Author Contributions

AC wrote the manuscript, designed the experiments, and performed WASPLab automation analyses. GR performed WASPLab automation analyses. YC performed population analysis profiles experiments. DB provided NARA clinical isolates and reviewed the manuscript. NV and JS reviewed and revised the manuscript.

### Conflict of Interest

The authors declare that the research was conducted in the absence of any commercial or financial relationships that could be construed as a potential conflict of interest.
